# Growth and Phytochemistry of *Cymbopogon citratus* Stapf Inoculated with Plant Growth-Promoting Bacteria under Different Lead Levels

**DOI:** 10.3390/plants13070944

**Published:** 2024-03-25

**Authors:** Rayane Monique Sete da Cruz, Henrique Ferreira, Jonas Marcelo Jaski, Marcelo Coelho Esperança Vieira, Mariana Moraes Pinc, Silvia Graciele Hülse de Souza, Odair Alberton

**Affiliations:** 1 Biochemistry and Microbiology Department, Biosciences Institute of Rio Claro, Paulista State University (UNESP), Rio Claro 13506-900, Brazil; rayanesete@hotmail.com; 2 Postgraduate Program in Biotechnology Applied to Agriculture, Universidade Paranaense (UNIPAR), Umuarama 87502-210, Brazil; henriqueferreira@unesp.br (H.F.); mariana.pinc@edu.unipar.br (M.M.P.); silviahulse@prof.unipar.br (S.G.H.d.S.); 3 Agronomy Department, Centro Universitário Ingá—Uningá, Maringá 87035-510, Brazil; jonasmjaski@gmail.com; 4 Postgraduate Program in Medicinal Plants and Herbal Medicines in Basic Health Care, Universidade Paranaense (UNIPAR), Umuarama 87502-210, Brazil; marcelo.esperanca@edu.unipar.br

**Keywords:** PGPB, rhizosphere, phytoremediation, soil rehabilitation, lemongrass, essential oil

## Abstract

This study aimed to investigate the phytochemistry of lemongrass (*Cymbopogon citratus)* inoculated with *Azospirillum brasilense* and grown in lead (Pb)-contaminated soil to assess its responses to inoculation under different Pb levels. The experimental design was completely randomized in a 2 × 5 factorial scheme: two levels of *A. brasilense* (absence or presence) and five Pb levels. After four months of treatment, the following were analyzed: total and reducing sugars, total phenolic content, flavonoids, antioxidant activity, antioxidant enzymes, proline, and essential oil (EO) content and composition. Soil Pb levels and *A. brasilense* inoculation affected phytochemicals in lemongrass plants. *Azospirillum* inoculation reduced total sugars in the roots at all soil Pb levels, while increasing Pb levels favored a rise in sugar contents. There was an increase in flavonoid content in treatments associated with Pb and inoculated with *A. brasilense*. Antioxidant capacity was lower at lower Pb levels, regardless of bacterial inoculation. Enzymatic response was mainly affected by Pb concentrations between 50 and 100 mg kg^−1^ soil. EO content was influenced by soil Pb levels, with higher EO production at 500 mg Pb kg^−1^ soil and without *A. brasilense* inoculation. Overall, lemongrass cultivation in Pb-contaminated areas can be an alternative to phytoremediation and EO production for the industry.

## 1. Introduction

Since the beginning of industrialization, a vast amount of organic pollutants (hydrocarbons, volatile organic compounds, and solvents) and inorganic pollutants (heavy metals) have been released into the environment [1]. Mining is the primary source of heavy metal contamination in soil, causing direct or indirect harm to plants and humans [2]. Among these pollutants, lead (Pb), considered a heavy metal, poses a significant risk due to its high toxicity and mutagenic potential [3,4]. 

In plants, Pb has negative effects on growth and development, impairing and altering the production of active compounds [5]. High levels of Pb in plants can impair chloroplast function, inhibiting chlorophyll biosynthesis, CO_2_ fixation, and the assembly of pigment-protein complexes in photosystems [6,7]. Pb-induced stress primarily damages the oxygen-evolving complex located in the photosystem II [8].

Pb toxicity leads to root growth inhibition, stunted plant growth resulting in chlorosis, and the disruption of various plant activities and properties, including antioxidant systems, respiration, mineral nutrition, membrane structure and properties, and gene expression [9]. As Pb-contaminated areas often become unsuitable for cultivation of food crops, growing plants known as phytoremediators stands out as an environmentally sustainable approach to remove heavy metals from the soil, retaining them in the aboveground or root biomass [10]. This allows for reclamation of contaminated areas for the cultivation of medicinal and aromatic plants, potentially generating a marketable end product, such as essential oil—EO [1,11]. 

Lemongrass, *Cymbopogon citratus* (D.C.) Stapf, is a plant widely used for phytoremediation due to its resistance to different heavy metals [12]. The commercial interest in its cultivation is primarily related to the cosmetics and perfumery industries, thanks to its EO [13], mainly composed of citral, which has two geometric isomers, geranial and neral, with a characteristic lemon scent [14]. Additionally, *C. citratus* contains minerals, vitamins, and bioactive compounds (alkaloids, terpenoids, flavonoids, phenols, saponins, and tannins) responsible for its pharmacological properties (antioxidant, antifungal, anticancer, antihypertensive, antidiabetic, and anxiolytic) [15,16]. Moreover, amid the global COVID-19 pandemic caused by the SARS-CoV-2 virus, the need for bioactive food ingredients has increased, as they stimulate the immune system, and natural polyphenols are reported as potential inhibitors of the main protease of COVID-19 [17], with the use of *C. citratus* being studied in the prevention, treatment, and control of the virus [18]. 

Plant metabolic responses are influenced by various factors, including the availability of heavy metals in the soil [5]. An alternative to establishing stable conditions for better plant development is the use of plant growth-promoting bacteria (PGPB), such as *Azospirillum brasilense* [19]. Bacteria of the *Azospirillum* genus can associate with the plant’s rhizosphere in external colonization or endophytically [19,20]. These bacteria promote plant growth through mechanisms such as amino acid biosynthesis and release, indole-acetic acid, cytokinins, gibberellins, and other polyamines, which enhance root growth and, consequently, improve water and nutrient absorption by plants [20,21]. Additionally, these microorganisms are capable of inducing the synthesis of antioxidant enzymes, reducing the deleterious effects of reactive oxygen species (ROS), and promoting greater root elongation, consequently improving the photosynthetic rate [22,23]. Plants under stress conditions have increased EO content, since oil production is a plant defense mechanism; *A. brasilense* reduces oxidative stress, which may result in reduced EO content [24].

Plants have developed mechanisms to alleviate heavy metal toxicity and survive in polluted soils, with one mechanism being the elimination of ROS by increasing the activity of antioxidant enzymes [25]. According to Basu et al. [26], this defense system includes enzymatic antioxidants such as catalase (CAT), superoxide dismutase (SOD), and ascorbate peroxidase (APX), as well as non-enzymatic antioxidants. These enzymes are involved in detoxification of oxygen radicals and can be induced by stress caused by high Pb concentrations in contaminated soils [27,28].

Studies have suggested that induction of antioxidant responses is an adaptive mechanism in plants to counter the oxidative stress of Pb accumulation. Enzymes such as superoxide SOD and peroxidase (POD) demonstrate increased activity, confirming that the antioxidant system can play a crucial role in mitigating Pb toxicity [28,29,30]. The activity of enzymes like SOD, ascorbate peroxidases (APX), and glutathione peroxidase (GPX) can be increased with inoculation of symbiotic microorganisms, enhancing the alleviation of Pb toxicity by eliminating reactive oxygen species (ROS) and reducing Pb concentrations in leaves [31,32].

In this context, this study aimed to investigate the growth and phytochemical responses of *C. citratus* inoculated with *A. brasilense* under different Pb levels, as well as to evaluate its responses to PGPB inoculation at different soil Pb levels.

## 2. Material and Methods

### 2.1. Experimental Design

The soil used in the experiment was characterized as Dark Red Latosol with medium texture and was collected from the experimental farm of the Universidade Paranaense (UNIPAR), Umuarama-PR, Brazil (Latitude: 23°45′51″ S, Longitude: 53°19′6″ W), at a depth of 0 to 20 cm. For chemical characterization, a soil sample was sent to the Laboratory of Soil Fertility located in Umuarama, PR (Table 1).

The experimental unit consisted of a polyethylene pot with a capacity for 3 L of soil. The soil was sieved through a 4 mm mesh and sterilized in an autoclave for 1 h at 120 °C, twice, with a 24 h interval, and allowed to cool for three days before setting up the experiment. Young lemongrass seedlings, approximately 20 cm in height, were collected from the medicinal garden of the UNIPAR and washed with running water. Two disinfected seedlings were transplanted into each pot after being previously disinfected in 70% alcohol for one minute. 

The experimental design used was completely randomized in a 2 × 5 factorial scheme: two levels of *A. brasilense* (absence or presence [1 mL plant^−1^]), strains Ab-V5 and Ab-V6, which form a commercial and registered inoculate used in Brazil [23,24] and five levels of lead (Pb), totaling sixty experimental units in ten treatments with six replications conducted in a greenhouse. The treatments were as follows:

Treatment 1: soil autoclaved + 0 Pb (mg Pb kg^−1^ of soil)

Treatment 2: soil autoclaved + 50 Pb (mg Pb kg^−1^ of soil)

Treatment 3: soil autoclaved + 100 Pb (mg Pb kg^−1^ of soil)

Treatment 4: soil autoclaved + 300 Pb (mg Pb kg^−1^ of soil)

Treatment 5: soil autoclaved + 500 Pb (mg Pb kg^−1^ of soil)

Treatment 6: soil autoclaved + *A. brasilense* + 0 Pb (mg Pb kg^−1^ of soil)

Treatment 7: soil autoclaved + *A. brasilense* + 50 Pb (mg Pb kg^−1^ of soil)

Treatment 8: soil autoclaved + *A. brasilense* + 100 Pb (mg Pb kg^−1^ of soil)

Treatment 9: soil autoclaved + *A. brasilense* + 300 Pb (mg Pb kg^−1^ of soil)

Treatment 10: soil autoclaved + *A. brasilense* + 500 Pb (mg Pb kg^−1^ of soil)

All treatments were irrigated every two days for a period of four months with half-strength solution developed by Hoagland and Arnon [34].

### 2.2. Determination of Total and Reducing Sugars

Total sugars (glucose, fructose, mannose, and sucrose) were quantified using the phenol–sulfuric acid method by reading at 540 nm on the spectrophotometer [35]. Reducing sugars were quantified using the dinitrosalicylic acid (DNS) method adapted for microplates, and the samples were read at 490 nm [36]. A calibration curve was established using glucose as a standard. Both total- and reducing-sugar quantifications were performed with three biological replicates, in triplicate.

### 2.3. Determination of Total Phenolic Content, Flavonoids and DPPH Antioxidant Activity

Total phenolics were determined colorimetrically using the Folin–Ciocalteu reagent, as described by [37], with readings at 760 nm (R^2^: 0.9962). The total flavonoid content was also determined spectrophotometrically at 425 nm (R^2^: 0.9917), using a method described by [38] based on the formation of an aluminum-flavonoid complex. The antioxidant activity of fresh plant-leaf extracts and standard antioxidants was evaluated based on the DPPH (2,2-diphenyl-1-picrylhydrazyl) free-radical scavenging effect, measured spectrophotometrically at 515 nm [39]. All analyses were performed with three biological replicates, in triplicate.

### 2.4. Antioxidant Enzymes

Fresh plant tissues were macerated in liquid nitrogen and then approximately 0.3 g of samples was homogenized in 1.5 mL of 200 mM potassium phosphate buffer (pH 7.8) containing 10 mM EDTA, 200 mM ascorbic acid, and 10% polyvinylpyrrolidone (PVPP), using a mortar and pestle. The homogenate was centrifuged at 16,128× *g*-force for 20 min at 4 °C, and the supernatant was collected and stored in an ultra-freezer (−80 °C) until analysis. The extracts were used to test for the antioxidant enzyme superoxide dismutase (SOD), catalase (CAT), and ascorbate peroxidase (APX). All assays were performed with three biological replicates, in triplicate [40].

#### 2.4.1. Superoxide Dismutase (SOD, EC 1.15.1.1)

The SOD activity was determined by its ability to inhibit reduction of nitroblue tetrazolium (NBT) by superoxide, forming blue formazan [41]. The reaction medium (1 mL) consisted of 50 μL of the crude sample extract, 50 mM KPO_4_ buffer (pH 7.8), 13 mM methionine, 0.1 μM EDTA, 75 μM NBT, and 2 μM riboflavin. The SOD activity was determined by spectrophotometry (560 nm) and expressed as U SOD g^−1^ FW min^−1^, where one unit of SOD activity (U) was defined as the amount of enzyme required to inhibit 50% of NBT reduction. 

#### 2.4.2. Catalase (CAT, EC 1.11.1.6)

The CAT activity was determined according to Havir and McHale [42]. The reaction medium (1 mL) consisted of 50 μL of the crude sample extract, 200 mM KPO_4_ buffer (pH 7.0), and 20 mM H_2_O_2_. The consumption of H_2_O_2_ was used to measure CAT activity by spectrophotometry (240 nm) for 1 min and then quantified using the molar extinction coefficient of 36 M^−1^ cm^−1^ [43]. The CAT activity was expressed as μmol H_2_O_2_ g^−1^ FW min^−1^.

#### 2.4.3. Ascorbate Peroxidase (APX, EC 1.11.1.11)

The reduction of H_2_O_2_ to H_2_O oxidizing ascorbic acid is catalyzed by ascorbate peroxidase (APX). The method proposed by Nakano and Asada [44] was used to determine APX activity. The reaction medium (1 mL) consisted of 50 μL of the crude sample extract, 50 mM KPO_4_ buffer (pH 7.0), 10 mM ascorbic acid, and 1 mM H_2_O_2_. APX activity was determined by H_2_O_2_ degradation monitored through spectrophotometry (290 nm) for 1 min and quantified using the molar extinction coefficient of 2.8 mM^−1^ cm^−1^. APX activity was expressed as μmol ascorbic acid g^−1^ FW min^−1^.

### 2.5. Proline 

Proline content was determined following the method proposed by Bates et al. [45]. Free proline contents in plant shoots were determined using fresh leaves (0.5 g), which were crushed with liquid nitrogen and mixed with 5 mL of a 3% sulfosalicylic acid solution. After centrifugation for 10 min at 16,128× *g*-force, 2 mL of the resulting filtrate was combined with 2 mL of ninhydrin and 2 mL of glacial acetic acid in a test tube. The mixture was then heated in a water bath at 100 °C for 1 h and cooled to room temperature. Afterward, 4 mL of toluene was used to extract the mixture, and the absorbance was measured at 520 nm. This assay was conducted with five biological replicates, each performed in triplicate, and the proline content was calculated using a pre-established proline standard curve (R^2^: 0.9958).

### 2.6. Essential oil Extraction and Yield Evaluation 

The essential oil (EO) was extracted by hydrodistillation using a modified Clevenger apparatus for 3 h, according to Cruz et al. [24]. After extraction, it was transferred to amber bottles and the solvent was allowed to evaporate, to calculate the content (m/m %), considering the plant mass versus the EO mass. The EO was stored in a freezer (−20 °C) until the chemical characterization of the EO.

### 2.7. Chemical Identification of Essential Oil by GC/MS

The EO was chemically identified using gas chromatography GC-MS QP 2010 SE (Shimadzu, Kyoto, Japan). Ten μL of the samples was diluted in 1000 μL of anhydrous dichloromethane before being injected into an SH-RTx-5MS column (Shimadzu, 5% phenylmethyl siloxane, 30 m × 0.25 mm id, 0.25 μm) using an autosampler (Shimadzu AOC-20i). Helium was used as carrier gas at a flow rate of 1.0 mL per min, with a split ratio of 2:1 and a sample injection amount of 1 μL. The column temperature was initially programmed at 40 °C, increasing by 8 °C per min to a final temperature of 300 °C. The injector and GC-MS interface temperatures were maintained at 250 °C. Mass spectra were recorded at 70 eV with a mass range of m/z 50 to 550 amu. The chemical compounds in the EO were identified based on library and GC–MS Postrun Analysis software v 2.72.

### 2.8. Statistical Analysis

Data were subjected to analysis of variance (ANOVA), with means being compared by the Duncan’s test (*p* ≤ 0.05) through the SPSS version 22.0 statistical program for Windows (SPSS Inc., Chicago, IL, USA). Principal component analysis (PCA) was performed to discriminate EO composition as a function of each treatment. All variables were analyzed using the Statistica v 13.0 software [46].

## 3. Results and Discussion

### 3.1. Primary and Secondary Metabolites

The total sugar content of the shoot in T6 (presence of *A. brasilense* and 0 Pb) was 788.39 mg g^−1^ of fresh mass, which was approximately 76.60% higher than in T1 (control). Therefore, the bacteria can increase the production of total sugars in leaves (Table 2). Such an increase may have occurred because carbohydrate reserves are used to provide the energy required to maintain the association with diazotrophic bacteria [47,48].

As for the roots, total sugar contents were higher in T3 (no bacteria and 100 Pb) at 4714.73 mg g^−1^ fresh mass. This result was 96.7% higher than the average of the treatments using inoculation with *A. brasilense*. Hence, plants inoculated with *A. brasilense* had lower total sugar contents in the roots at all Pb levels in the soil. This outcome may be due to its effects in alleviating the stress, caused by heavy metals, in plant metabolism. However, plants grown in Pb-contaminated soils without the presence of *Azospirillum* bacteria produced higher levels of total sugars in the roots, probably due to stress caused by Pb on their metabolism. Reducing sugars were minimally affected by Pb levels in the soil, with a higher production of reducing sugars in the roots in T9 (bacteria present and 300 Pb), reaching 2764.81 µg g^−1^ fresh mass. 

The higher contents of total sugars due to Pb may be related to their function in plants. High sugar concentrations are known to promote carbohydrate storage, while low levels stimulate photosynthesis and reserve mobilization and export, directly influencing the source–sink relationships in plants [49,50]. Sugars can also act as an osmoprotectant in regulating osmotic adjustment, providing membrane protection and eliminating toxic reactive oxygen species (ROS) under various stress conditions, including Pb stress [51,52]. 

Low concentrations of soluble sugars, such as glucose and sucrose, under stress conditions, also stimulate the activity of antioxidant enzymes such as peroxidase (APX), catalase (CAT), and superoxide dismutase (SOD), which are essential enzymatic systems responsible for cellular homeostasis and detoxification [53]. 

The production of flavonoids in plant shoots (Table 3) was increased in T10 (674.06 mg g^−1^ of fresh mass), while decreasing by approximately 80.32% in T6 and 77.43% in T1. The same trend was observed in the roots, where plants produced higher amounts of flavonoids in T10 (767.54 mg g^−1^ of fresh mass). Higher Pb concentrations in the soil increase the production of flavonoids due to the need for protection against oxidizing agents [54,55]. 

Overall, all treatments associated with Pb and inoculated with *A. brasilense* showed higher productions of flavonoids; therefore, *Azospirillum* inoculation induced a greater formation of flavonoids both in the roots and shoots of plants. Flavonoids play an important antioxidant role, especially in complementing the action of antioxidant enzymes when they are inactivated or have insufficient activity to counteract ROS during stress conditions [56,57,58]. Highly hydroxylated flavonoids are induced by abiotic stress, and the presence of an additional free hydroxyl (-OH) on the C-3′ of the B ring contributes to a stronger elimination capacity under these conditions [59,60].

Another class of secondary metabolites related to plant defense responses against environmental stressors is phenolic compounds, which, due to their stable intermediate radicals, prevent lipid oxidation [61]. In this sense, the treatment that presented the highest number of phenolic compounds in plant shoots was T5, as it was under greater stress. In contrast, in the roots, T10 exhibited the highest amount of phenolic compounds, as plants perceive the initial inoculation with *A. brasilense* as a stress factor, along with a high Pb dosage [62]. 

According to the antioxidant activity (DPPH) analysis, treatments T1, T2 (0 and 50 Pb with no bacteria inoculation), T6, and T7 (0 and 50 Pb with no bacteria present) did not significantly differ from each other, showing higher antioxidant capacity than the other treatments. Lower Pb levels provided higher antioxidant activity regardless of the presence of the PGPB. However, *A. brasilense* is known for inducing the production of antioxidant enzymes and reducing deleterious effects from reactive oxygen species (ROS) [22,24].

### 3.2. Response of Antioxidant Enzymes to Lead Stress

**Figure 1:**
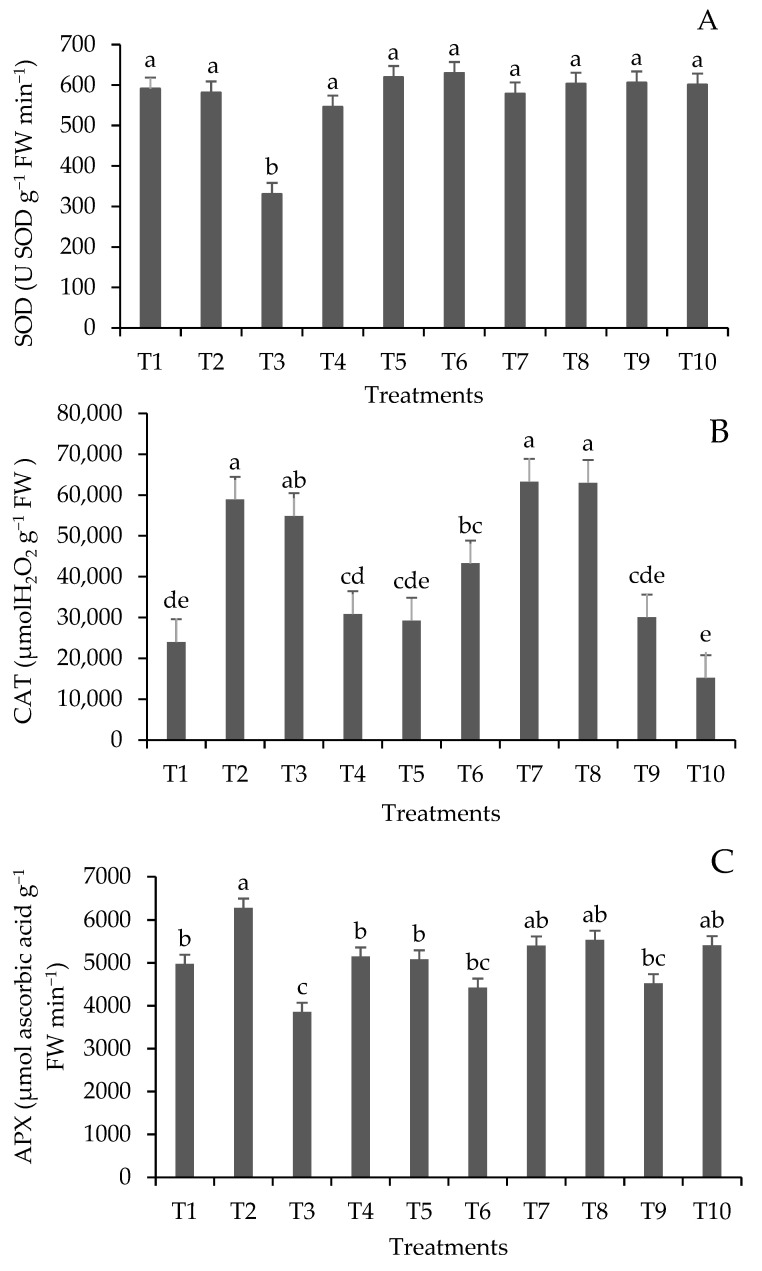
Antioxidant enzyme activity of superoxide dismutase (SOD) (**A**), catalase (CAT) (**B**), and ascorbate peroxidase (APX) (**C**) of lemongrass plants inoculated with *Azospirillum*^®^ and grown in soils under different lead levels. Mean values (n = 6 ± standard error); different letters in the same column differ significantly according to the Duncan test (*p* ≤ 0.05). T1: control (autoclaved soil); T2: autoclaved soil + 50 mg Pb kg^−1^; T3: autoclaved soil + 100 mg Pb kg^−1^; T4: autoclaved soil + 300 mg Pb kg^−1^; T5: autoclaved soil + 500 mg Pb kg^−1^; T6: autoclaved soil + *A. brasilense*; T7: autoclaved soil + *A. brasilense* + 50 mg Pb kg^−1^; T8: autoclaved soil + *A. brasilense* + 100 mg Pb kg^−1^; T9: autoclaved soil + *A. brasilense* + 300 mg Pb kg^−1^; T10: autoclaved soil + *A. brasilense* + 500 mg Pb kg^−1^.

The capacity of antioxidant systems to detoxify ROS is closely related to plant tolerance to heavy metals [63]. In recent years, many plant species have been identified as accumulators, with the ability to accumulate heavy metals without impacting their growth and development [32,64]. The enzyme SOD, which constitutes the first line of defense against ROS and is responsible for dismutation of superoxide radicals (O_2_^−^) into hydrogen peroxide (H_2_O_2_), showed a significant result (*p* ≤ 0.05) only in treatment T3 (Figure 1). 

CAT, an enzyme that degrades H_2_O_2_ without consuming the reducing equivalents, and is responsible for peroxide removal in excess due to its low affinity for H_2_O_2_, showed higher activity in T2, T3, T7, and T8, which are treatments with 50 and 100 mg Pb kg^−1^ soil with and without inoculation of *A. brasilense*. 

The response of APX, a key enzyme in the glutathione–ascorbate cycle, which reduces H_2_O_2_ to H_2_O using ascorbate as an electron donor with the concomitant generation of dehydroascorbate, was prominent in T2, with 6282.96 μmol of ascorbic acid g^−1^ FW min^−1^. The observed pattern of enzymatic response showed that Pb concentrations between 50 and 100 mg kg^−1^ induce the synthesis of antioxidant enzymes in response to Pb stress. Inoculation with *A. brasilense* did not significantly affect the synthesis of the studied enzymes, although Wang et al. [65] reported that bacteria can secrete antioxidant enzymes to prevent oxidative damage.

**Figure 2:**
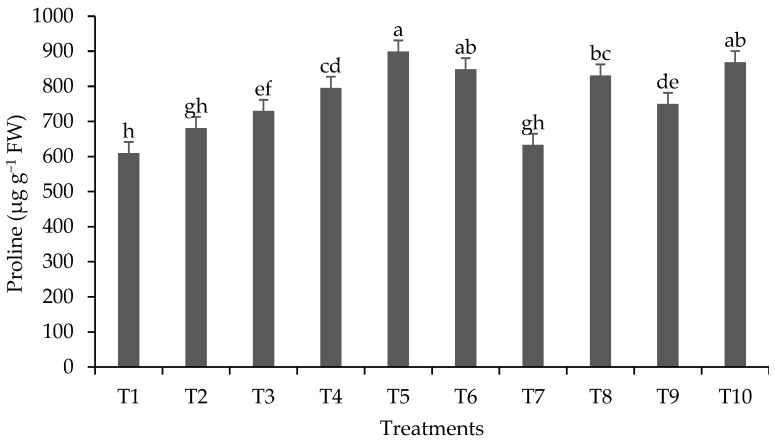
Proline content (µg g^−1^ fresh leaves (FW)) in shoots of lemongrass plants inoculated with *Azospirillum*^®^ and grown in soils under different lead levels. Mean values (n = 6 ± standard error); different letters in the same column differ significantly according to the Duncan test (*p* ≤ 0.05). T1: control (autoclaved soil); T2: autoclaved soil + 50 mg Pb kg^−1^; T3: autoclaved soil + 100 mg Pb kg^−1^; T4: autoclaved soil + 300 mg Pb kg^−1^; T5: autoclaved soil + 500 mg Pb kg^−1^; T6: autoclaved soil + *A. brasilense*; T7: autoclaved soil + *A. brasilense* + 50 mg Pb kg^−1^; T8: autoclaved soil + *A. brasilense* + 100 mg Pb kg^−1^; T9: autoclaved soil + *A. brasilense* + 300 mg Pb kg^−1^; T10: autoclaved soil + *A. brasilense* + 500 mg Pb kg^−1^.

Regarding proline content (Figure 2), the highest accumulation occurred in T5 (899.18 µg g^−1^ fresh mass). In metal-accumulator plants like lemongrass, an amino acid proline synthesis mechanism is activated as a defense mechanism, which not only mitigates oxidative stress but also restores the osmotic balance of plant cells [66]. In the case of treatments T7 and T9, inoculated with *A. brasilense*, proline formation was lower compared to treatments with Pb and without inoculation, indicating that *A. brasilense* may have beneficial effects in reducing Pb stress in lemongrass. The bacterium has been shown to assist in abiotic stress tolerance, alleviating stress caused by heavy metals [19,67]. Among the mechanisms activated for stress relief is induced systemic tolerance (IST), which is mediated by antioxidants, phytohormone production, osmotic adjustment, and defense strategies such as pathogenesis-related (PR) gene expression [22].

### 3.3. Extraction, Evaluation of EO Content and Chemical Identification by GC/MS

**Figure 3:**
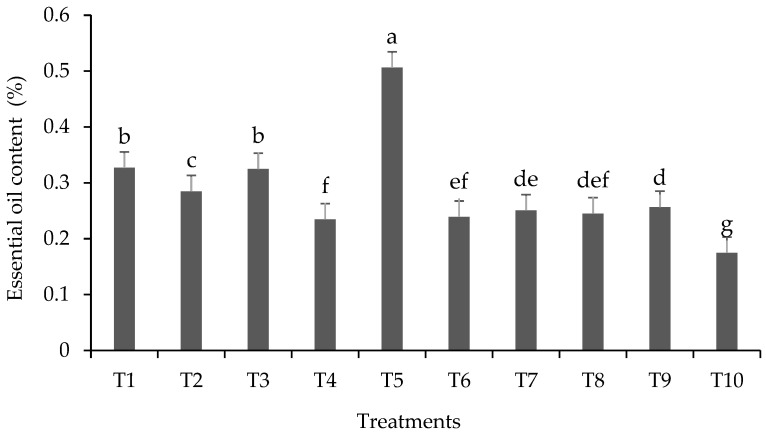
Essential oil content (%) of lemongrass plants inoculated with *Azospirillum*^®^ and grown in soils under different lead levels. Mean values (n = 3 ± standard error); different letters in the same column differ significantly according to the Duncan test (*p* ≤ 0.05). T1: control (autoclaved soil); T2: autoclaved soil + 50 mg Pb kg^−1^; T3: autoclaved soil + 100 mg Pb kg^−1^; T4: autoclaved soil + 300 mg Pb kg^−1^; T5: autoclaved soil + 500 mg Pb kg^−1^; T6: autoclaved soil + *A. brasilense*; T7: autoclaved soil + *A. brasilense* + 50 mg Pb kg^−1^; T8: autoclaved soil + *A. brasilense* + 100 mg Pb kg^−1^; T9: autoclaved soil + *A. brasilense* + 300 mg Pb kg^−1^; T10: autoclaved soil + *A. brasilense* + 500 mg Pb kg^−1^.

The content of lemongrass EO ranged from 0.17 to 0.50% (Figure 3). These findings are similar to those found in the literature: variations between 0.21 and 0.69% [5], 0.20 to 0.75% [24,68] and 0.20 to 0.76% [69]. The highest EO content was observed in T5 (~0.5%), which received the highest Pb concentration in the soil and had no *A. brasilense* inoculation. Overall, treatments with *A. brasilense* inoculation showed lower EO levels; therefore, Pb stress increased EO production in non-inoculated plants, as stressed plants tend to produce higher EO concentrations. In medicinal plants, EO content is known to vary due to numerous biotic and abiotic factors, including levels of chemical elements in the soil, so the stress caused by these elements can increase EO production in plants [70,71]. In the present study, *A. brasilense* inoculation may have alleviated plant stress caused by Pb, resulting in lower EO production in inoculated plants compared to non-inoculated ones. 

The chemical characterization of EO by GC/MS (Table 4) revealed the presence of 21 components, predominantly oxygenated monoterpenes, which greatly contribute to its fragrance [14]. The compounds neral (22.79% to 66.79%) and geranial (16.95% to 51.01%) were the main components. Extraction from fresh leaves allows for identification of the neral and geranial isomers (citral Z and E, respectively), which have deeply embedded structures that are difficult to access in dried leaves [24,68]. 

**Figure 4:**
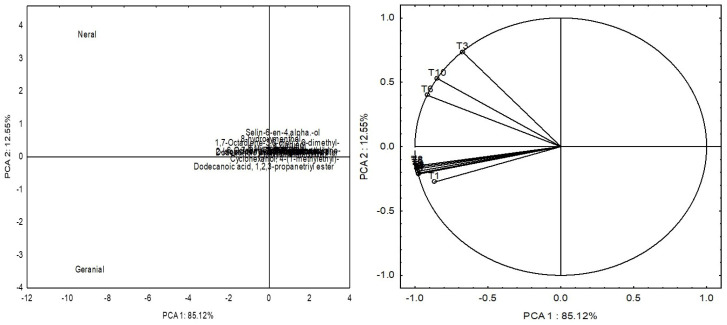
Biplot representation of a PCA (principal component analysis) performed on essential oils of the lemon of lemongrass plants inoculated with *Azospirillum*^®^ and grown in soils under different lead levels. T1: control (autoclaved soil); T2: autoclaved soil + 50 mg Pb kg^−1^; T3: autoclaved soil + 100 mg Pb kg^−1^; T4: autoclaved soil + 300 mg Pb kg^−1^; T5: autoclaved soil + 500 mg Pb kg^−1^; T6: autoclaved soil + *A. brasilense*; T7: autoclaved soil + *A. brasilense* + 50 mg Pb kg^−1^; T8: autoclaved soil + *A. brasilense* + 100 mg Pb kg^−1^; T9: autoclaved soil + *A. brasilense* + 300 mg Pb kg^−1^; T10: autoclaved soil + *A. brasilense* + 500 mg Pb kg^−1^.

Principal component analysis (PCA) based on EO data from the ten different treatments showed that the components (PC1 and PC2) explained 85.12% and 12.55% of the variability among the chemical constituents of EO (Table 4). The PCA factorial loading graph (Figure 4) of EO constituents clearly separates treatments T3, T6, and T10, directly influencing the neral component, from the other treatments (T1, T2, T4, T5, T7, T8, and T9), which were highly correlated with the production of geranial. The concentration of neral and geranial behaved differently in relation to the Pb concentration in the soil [5], and the inoculation of *A. brasilense* also influences this relationship between neral and geranial; this relationship between microorganisms and EO compound production is still not well understood [24]. 

According to Bernstein et al. [72], hydrodistillation for EO extraction allows for its extraction with nearly imperceptible levels of heavy metals, enabling its use in the food, pharmaceutical, and perfume industries, as citral is a raw material for production of ionone, vitamin A, and β-carotene. In addition to having a characteristic lemon scent, it can be used in various applications [73]. 

According to our findings, lemongrass can be grown in Pb-contaminated environments, favoring soil phytoremediation, and inoculation with *A. brasilense* can help reduce chemical fertilizations, promoting production system sustainability. This is expected to pave the way for further research to identify metabolic pathways and influence of microorganisms involved in EO production, as well as the phytoremediation of contaminated soils. Our perspective is that lemongrass could to be used as a biotechnological tool for the eco-restoration of Pb-contaminated sites, bringing profitability to farmers.

## 4. Conclusions

*A. brasilense* inoculation was effective in reducing high Pb-concentration-related stress in the soil. Inoculated plants had lower sugar contents in the roots, even in the presence of Pb in the soil, and showed increased flavonoid formation. Enzymatic responses were mainly affected by Pb concentrations of between 50 and 100 mg kg^–1^ in the soil. 

EO content increased in plants grown in the soil with the highest Pb levels and without inoculation with *A. brasilense*. However, inoculated plants did not exhibit an increase in EO content, possibly due to the reduction in Pb-induced stress, as stressed plants typically produce higher EO concentrations. In conclusion, cultivating lemongrass in Pb-contaminated areas seems to be a promising alternative for EO production, as it did not significantly alter its composition. Moreover, *A. brasilense* inoculation proved to be beneficial in mitigating stress caused by high Pb concentrations in contaminated soils

## Figures and Tables

**Table 1:** Chemical properties of the soil used in the experiment.

	pH (CaCl_2_)	P	C	Al^3+^	H^+^ + Al^3+^	Ca^2+^	Mg^2+^	K^+^	SB	CEC	BS
		mg dm^−3^	g dm^−3^	Cmol_c_ dm^−3^							%
Soil	6.07	1.54	4.09	0.00	1.89	1.38	0.75	0.05	2.18	4.07	53.52
Ref ^1^	3.8–6.6	16–24	0.8–15.9	-	0.6–5.0	0.3–7.2	0.3–3.3	0.1–0.7	-	2.2–12.5	-

SB = sum of bases; CEC = cation exchange capacity; BS% = base saturation. Methods: P, K—extracted by Mehlich-I; Ca, Mg and Al—extracted by KCl, 1 mol L^−1^; C—Dichromate/colorimetric. ^1^ Source: [33].

**Table 2:** Total sugars in shoot (mg g^−1^ fresh mass) (TSS), total sugars in roots (mg g^−1^ fresh mass) (TSR), reducing sugars in shoot (µg g^−1^ fresh mass) (RSS), and reducing sugars in roots (µg g^−1^ fresh mass) (RSR) of lemongrass plants inoculated with *Azospirillum*^®^ and grown in soils under different lead levels.

Treatment	TSS	TSR	RSS	RSR
T1	184.56 ± 7.75 d	3849.19 ± 423.70 b	2337.80 ± 43.32 cd	2419.50 ± 45.82 b
T2	108.66 ± 1.11 e	2676.01 ± 123.40 c	2780.62 ± 65.93 a	2178.07 ± 101.15 c
T3	119.52 ± 3.24 e	4714.73 ± 329.25 a	2639.45 ± 69.21 ab	2183.84 ± 40.01 c
T4	472.14 ± 11.63 b	1254.81 ± 35.89 d	2595.69 ± 106.51 ab	2368.12 ± 62.52 bc
T5	117.85 ± 0.33 e	561.02 ± 68.17 e	2497.59 ± 20.95 bc	2389.64 ± 46.96 bc
T6	788.39 ± 51.84 a	238.93 ± 1.99 e	2524.99 ± 121.51 bc	2317.78 ± 44.04 bc
T7	125.56 ± 0.97 e	115.91 ± 1.11 e	2439.73 ± 86.55 bc	2415.42 ± 53.76 b
T8	243.07 ± 1.59 c	200.51 ± 4.41 e	2216.46 ± 55.18 d	2361.74 ± 97.46 bc
T9	106.13 ± 0.68 e	129.63 ± 0.82 e	2569.70 ± 70.05 abc	2764.81 ± 88.66 a
T10	216.50 ± 3.25 cd	105.30 ± 0.60 e	2474.95 ± 33.65 bc	2492.77 ± 43.43 b

Mean values (n = 6 ± standard error); different letters in the same column differ significantly according to the Duncan test (*p* ≤ 0.05). T1: control (autoclaved soil); T2: autoclaved soil + 50 mg Pb kg^−1^; T3: autoclaved soil + 100 mg Pb kg^−1^; T4: autoclaved soil + 300 mg Pb kg^−1^; T5: autoclaved soil + 500 mg Pb kg^−1^; T6: autoclaved soil + *A. brasilense*; T7: autoclaved soil + *A. brasilense* + 50 mg Pb kg^−1^; T8: autoclaved soil + *A. brasilense* + 100 mg Pb kg^−1^; T9: autoclaved soil + *A. brasilense* + 300 mg Pb kg^−1^; T10: autoclaved soil + *A. brasilense* + 500 mg Pb kg^−1^.

**Table 3:** Flavonoids in shoot (mg g−1 fresh mass) (FlavS), flavonoids in roots (mg g−1 fresh mass) (FlavR), phenolic compounds in shoot (µg g−1 fresh mass) (PhenS), phenolic compounds in roots (µg g−1 fresh mass) (PhenR) and antioxidant activity (DPPH; %) in shoot of lemongrass plants inoculated with *Azospirillum*^®^ and grown in soils under different lead levels.

Treatment	FlavS	FlavR	PhenS	PhenR	DPPH
T1	152.15 ± 5.81 f	32.34 ± 2.45 f	1207.59 ± 20.61 e	262.64 ± 4.97 j	36.91 ± 0.74 a
T2	225.12 ± 1.16 e	40.12 ± 1.52 ef	1325.29 ± 34.80 d	309.28 ± 11.57 i	34.69 ± 0.07 a
T3	224.81 ± 1.53 e	25.12 ± 4.38 f	1958.21 ± 7.87 c	348.88 ± 11.26 h	20.13 ± 0.28 c
T4	236.78 ± 12.54 e	44.96 ± 2.51 ef	2479.73 ± 43.84 b	385.89 ± 3.56 g	12.70 ± 0.12 d
T5	382.69 ± 9.29 d	68.30 ± 10.16 e	2771.76 ± 50.72 a	511.74 ± 1.23 f	12.64 ± 0.22 d
T6	132.69 ± 1.34 g	434.81 ± 11.33 d	1284.57 ± 2.72 de	617.23 ± 2.39 e	35.50 ± 1.59 a
T7	234.51 ± 2.71 e	468.45 ± 27.29 c	1330.47 ± 41.42 d	722.12 ± 22.95 d	34.53 ± 1.38 a
T8	438.75 ± 2.29 c	584.72 ± 1.48 b	1351.20 ± 19.47 d	875.95 ± 13.61 c	15.07 ± 1.22 d
T9	492.54 ± 2.55 b	604.36 ± 1.29 b	1893.07 ± 33.93 c	1081.00 ± 11.06 b	13.07 ± 0.10 d
T10	674.06 ± 2.87 a	767.54 ± 1.24 a	1374.15 ± 28.82 d	2175.48 ± 2.96 a	28.21 ± 0.31 b

Mean values (n = 6 ± standard error); different letters in the same column differ significantly according to the Duncan test (*p* ≤ 0.05). T1: control (autoclaved soil); T2: autoclaved soil + 50 mg Pb kg^−1^; T3: autoclaved soil + 100 mg Pb kg^−1^; T4: autoclaved soil + 300 mg Pb kg^−1^; T5: autoclaved soil + 500 mg Pb kg^−1^; T6: autoclaved soil + *A. brasilense*; T7: autoclaved soil + *A. brasilense* + 50 mg Pb kg^−1^; T8: autoclaved soil + *A. brasilense* + 100 mg Pb kg^−1^; T9: autoclaved soil + *A. brasilense* + 300 mg Pb kg^−1^; T10: autoclaved soil + *A. brasilense* + 500 mg Pb kg^−1^.

**Table 4:** Chemical characterization (%) of the components of the essential oil of lemongrass plants inoculated with *Azospirillum*^®^ and grown in soils under different lead levels.

Peak	^1^ RI	Component	T1	T2	T3	T4	T5	T6	T7	T8	T9	T10
1	8.342	*β* Myrcene	2.46	3.79	8.04	4.73	6.63	3.66	7.31	8.05	4.87	1.79
2	14.568	Neral (Citral Z)	22.79	33.02	63.18	33.83	34.54	33.31	33.69	33.58	33.59	66.79
3	15.265	Geranial (Citral E)	35.7	48.02	t	50.44	47.03	49.31	50.79	44.74	51.01	16.95
4	15.550	Epoxy-linalooloxide	2.66	t	t	t	t	t	t	t	t	t
5	16.856	Dodecanoic acid. 2-hexen-1-yl ester	3.86	t	2.9	t	t	t	2.43	t	t	t
6	17.664	Cyclohexanol. 4-(1-methylethyl)-	5.97	t	t	t	t	t	t	t	t	t
7	42.681	Dodecanoic acid. 1.2.3-propanetriyl ester	19.87	t	t	t	t	t	t	t	t	t
8	8.280	methyl heptenone	t	0.34	t	t	t	t	t	t	t	t
9	10.908	cis-Myroxide	t	0.39	0.75	0.5	0.64	0.47	0.85	0.96	0.54	t
10	11.179	Linalool	t	0.53	0.98	0.51	0.6	t	t	0.56	t	0.51
11	12.202	6-Octenal. 7-methyl-3-methylene-	t	0.39	0.75	0.4	0.42	0.39	t	0.43	t	0.38
12	12.689	Isoneral	t	0.7	2.34	0.76	0.82	0.7	t	0.8	0.62	0.58
13	13.125	Isogeranial	t	0.89	2.68	1	1.12	0.92	0.85	1.07	0.91	0.85
14	15.802	2-Undecanone	t	0.3	0.52	0.3	t	0.38	t	0.36		0.28
15	16.947	2.7-Dimethyl-2.7-octanediol	t	2.41		2.34	2.3	2.96	t	3.19	2.5	2.16
16	17.783	8-hydroxymenthol	t	4.31	6.94	4.21	3.72	5.13	2.79	5.13	4.32	3.85
17	22.920	Selin-6-en-4.alpha.-ol	t	3.05	7.02	0.97	1.56	1.59	t	0.51	1.08	3.28
18	23.580	*α* Cadinol	t	1.16	3.64	t	0.6	0.59	t	t	t	1.31
19	15.602	1-Undecyne	t	t	t	t	t	0.27	t	0.24	t	t
20	15.675	2-Isopropenyl-5-methylhex-4-enal	t	t	t	t	t	0.31	t	0.35	t	t
21	20.070	2-Tridecanone	t	t	t	t	t	t	t	t	0.43	0.56
		Total	93.31	99.3	99.74	99.99	99.98	99.99	98.71	99.97	99.87	99.29

t: trace. ^1^ Identification based on retention index (RI). T1: control (autoclaved soil); T2: autoclaved soil + 50 mg Pb kg^−1^; T3: autoclaved soil + 100 mg Pb kg^−1^; T4: autoclaved soil + 300 mg Pb kg^−1^; T5: autoclaved soil + 500 mg Pb kg^−1^; T6: autoclaved soil + *A. brasilense*; T7: autoclaved soil + *A. brasilense* + 50 mg Pb kg^−1^; T8: autoclaved soil + *A. brasilense* + 100 mg Pb kg^−1^; T9: autoclaved soil + *A. brasilense* + 300 mg Pb kg^−1^; T10: autoclaved soil + *A. brasilense* + 500 mg Pb kg^−1^.

## Data Availability

Data will be made available on request.
